# The Hospital Position at Wakefield

**Published:** 1921-08-20

**Authors:** 


### The Hospital Position at Wakefield.

The financial difficulties of the Clayton Hospital, Wakefield, seem partly due to the fact that it possesses certain special gifts and endowments. Since it is asserted by those who know best that these gifts have led to an exaggerated idea of its resources, we can understand the once familiar point of view that the best asset of a voluntary hospital was to have a debt. This asset, at all events, is possessed by the Clayton Hospital, which, in the words of Mr. J. W. Walker, is "£12,000 to the bad"; that this fact should be less moving to many of the public than its possession of a few investments does not speak well for their sincerity. The present situation of hospitals has necessitated a new method of finance, in which the need for a secure annual income takes the first place. The workmen's contributions in Wakefield already are a great help, but they would help even more with a greater uniformity of encouragement from the employers. The hospital needs a new nurses' home and a new $x$-ray apparatus, but the provision of even these would be robbed of their effect if the income is not put upon a more satisfactory basis. The idea that any share of the Government grant will depend upon the raising of an equal sum (in annual revenue) is now general, and if this is made the basis of a new campaign in Wakefield an assured income from regular contributions may arise in a way which would not follow were an appeal issued for a special sum. The modern appeal is for income rather than donations. Consequently the plan of campaign has to differ also by creating a new organisation of regular subscribers, with or without the inducements offered by the Sussex or Oxford schemes. The Local Hospital Committee, being formed in relation to the Voluntary Hospitals' Commission, should see that any appeal which the Wakefield Hospital may make is directed to the right end—namely, an increase of annual income.

